# Consumption of Low-Calorie Sweeteners among U.S. Adults Is Associated with Higher Healthy Eating Index (HEI 2005) Scores and More Physical Activity

**DOI:** 10.3390/nu6104389

**Published:** 2014-10-17

**Authors:** Adam Drewnowski, Colin D. Rehm

**Affiliations:** Center for Public Health Nutrition, University of Washington, Box 353410; Seattle, WA 98195, USA; E-Mail: crehm@uw.edu

**Keywords:** low-calorie sweeteners, diet quality, Healthy Eating Index 2005, health behaviors

## Abstract

The possibility that low-calorie sweeteners (LCS) promote lower quality diets and, therefore, weight gain has been noted as a cause for concern. Data from a representative sample of 22,231 adults were obtained from five cycles of the National Health and Nutrition Examination Survey (1999–2008 NHANES). A single 24-hour recall was used to identify consumers of LCS beverages, foods and tabletop sweeteners. Diet quality was assessed using the Healthy Eating Index 2005 (HEI 2005) and its multiple subscores. Health behaviors of interest were physical activity, smoking and alcohol use. LCS consumers had higher HEI 2005 scores than did non-consumers, largely explained by better SoFAAS subscores (solid fats, added sugar and alcohol). LCS consumers had better HEI subscores for vegetables, whole grains and low-fat dairy, but worse subscores for saturated fat and sodium compared to non-consumers. Similar trends were observed for LCS beverages, tabletop LCS and LCS foods. Consumers of LCS were less likely to smoke and were more likely to engage in recreational physical activity. LCS use was associated with higher HEI 2005 scores, lower consumption of empty calories, less smoking and more physical activity.

## 1. Introduction

Replacing added sugars in beverages and foods with low-calorie sweeteners (LCS) is one way to manage body weight [1,2,3,4]. Overweight adults are more likely to consume LCS products than are normal-weight adults [5]. Combining LCS use with higher-quality diets and with more physical activity would be an even more comprehensive approach to weight control [6,7].

Based on existing data, the consumers of diet products in the U.S. are more likely to be non-Hispanic white women with higher education and incomes [8]. The consumption of diet beverages in the U.S. is higher among groups of higher socioeconomic status (SES), as is the consumption of bottled and tap water [9]. Diet quality, as measured by Healthy Eating Index (HEI 2005) scores, also tends to be higher among individuals of higher SES [10]. Physical activity and smoking follow similar socio-demographic trends [11,12].

Concerns about the use of LCS for weight control [4] have invoked the possibility that the use of sweet, yet non-caloric LCS products might confuse the body, provoke increased appetite for sweet foods, reduce overall diet quality and contribute to weight gain. The present hypothesis was that, to the contrary, LCS use would be associated with better-quality diets and with more positive health behaviors, after adjusting for covariates [6,13].

LCS consumers were stratified by product type: LCS beverages, tabletop LCS and LCS foods [14]. Although diet beverages are the category leader, tabletop LCS and LCS foods also play a role in weight control [15]. Diet quality was assessed using the Healthy Eating Index (HEI 2005), a tool developed by the U.S. Department of Agriculture to measure compliance with U.S. dietary recommendations and guidelines [16]. Among HEI subscores, the consumption of solid fats, alcohol and added sugars (SoFAAS subscore) was of particular interest. Any increase in added sugars consumption would be reflected in a less favorable SoFAAS subscore [5].

## 2. Experimental Section

### 2.1. Population Sample

The National Health and Nutrition Examination Survey (NHANES) provides data on dietary intakes and multiple health indicators for a nationally-representative sample of children and adults in the United States [17]. The present analyses used data from 5 NHANES cycles: 1999–2000, 2001–2002, 2003–2004, 2005–2006 and 2007–2008. Included in the analysis were data for 22,231 adults (≥20 years), who were not pregnant, for whom height and weight data were available and who completed a 24-h dietary recall. All study protocols for NHANES 1999–2008 were approved by the institutional review board at the National Center for Health Statistics [18], and informed consent was provided by all participants.

Dietary collection methods differed depending on the cycle of NHANES. NHANES 1999–2002 collected data using a computer-assisted dietary interview system (CADI), whereas the later cycles (NHANES 2003–2008) used the USDA Automated Multiple Pass Method, administered by trained interviewers, to obtain 24-h recalls. Respondents reported the types and amounts of all food and beverages consumed in the preceding 24-h, from midnight to midnight. The detailed methodology has been reported elsewhere [17,19]. Further, NHANES 1999–2002 collected only one 24-h recall, whereas the later cycles collected 2 recalls, one in person and one by telephone. The present analyses were based on the first 24-h recall. A single 24-h recall for a large population yields an unbiased estimate of the dietary patterns of populations. Participant characteristics, including age, gender, education and race/ethnicity, were self-reported. 

### 2.2. Classification of LCS Consumption by Product Category

The Food and Nutrition Database for Dietary Studies [20] used to calculate energy and nutrient intakes in NHANES does not formally code foods and beverages containing LCS. To develop a custom coding algorithm, we examined approximately 5700 items in the individual foods’ file to identify those foods and beverages that did contain LCS. Individual foods and beverages were queried based on the food description, energy density (kcal/100 g) and total and added sugars content (g) per average consumption report.

The most common LCS beverages were “soft drink, cola-type, sugar-free”, “soft drink, cola-type, decaffeinated, sugar-free”, “soft drink, fruit-flavored, sugar-free, caffeine free” and “fruit-flavored drink, made from powder, low calorie”. The LCS designation also included teas pre-sweetened with LCS. The most frequently used tabletop LCS were saccharin, sucralose and aspartame. Liquid LCS were also included in this category, but represented a very small number of consumption reports compared to powder-based LCS. Key LCS foods included yogurt, ice cream, grain-based desserts and candies. The level of detail in the food database did not permit for the evaluation of specific types of LCS (e.g., comparison of sucralose *vs*. saccharin).

NHANES participants were then assigned to 4 different categories of LCS consumers, namely: (1) consumers of LCS beverages (e.g., diet soft drinks, diet fruit drinks, diet iced tea and low-calorie energy drinks); (2) consumers of LCS foods (e.g., yogurt, ice cream, baked goods or candies); (3) consumers of tabletop LCS (e.g., sucralose, aspartame or saccharin); and (4) consumers of any LCS from any source (beverages, foods or tabletop).

### 2.3. Diet Quality Measures and Health Behaviors

Diet quality was assessed using the Healthy Eating Index (HEI 2005), a tool developed by the U.S. Department of Agriculture to measure compliance with dietary recommendations and guidelines. The HEI 2005 is a 12-component 100-point scale that assesses the adequacy and moderation components of the American diet. Higher scores are associated with better dietary compliance [10,16]. Physical activity was assessed through a self-reported questionnaire asking about various moderate and vigorous physical activities. Metabolic Equivalent of Tasks (MET) values were estimated from the physical activity questionnaire. Smoking status was categorized into: current, former or never, based on self-report. Alcohol use was assessed through both the 24-h recall and an alcohol use questionnaire, which was used to assess habitual alcohol use (e.g., average number of drinks per week).

### 2.4. Statistical Analysis

Descriptive analyses compared LCS consumers and non-consumers separately for each product category (beverage, tabletop, food). For all analyses, except those by age group, estimated proportions were adjusted for age group using direct standardization. Statistical heterogeneity was evaluated with a survey-weighted Wald test. In analyses adjusted for age group, gender and race/ethnicity a survey-weighted linear (for continuous outcomes like HEI 2005) or multinomial logistic (for categorical outcomes like physical activity) regression model was fit. The survey-weighted marginal means/proportions were then estimated, which can be considered estimates of the age, gender and race-adjusted means/proportions. Data analysis was conducted using Stata 11 (College Station, TX, United States). All analyses accounted for the complex survey-design of NHANES data and are representative of the U.S. population.

## 3. Results

The socio-demographic variables for any LCS consumers and non-consumers are shown in Table 1. LCS consumers were more likely to be older, female, non-Hispanic whites, born in the United States and with higher education and incomes. The effects of all of the socio-demographic variables were significant. The age range for peak LCS consumption was 45–74 years.

The socioeconomic gradient was strong: LCS were consumed by 17.3% of persons with a <9th grade education and by 37.4% of persons with a college education. LCS consumption almost doubled from the lowest to highest categories of the family income-to-poverty ratio.

**Table 1:** Age-adjusted proportions consuming any low-calorie sweetener (LCS) (and standard errors) by socio-demographic group, National Health and Nutrition Examination Survey (NHANES) 1999–2008.

	*n*	Any LCS	No LCS	*P*-difference
Total	22,231	30.0 (0.6)	70.0 (0.6)	-
Age group ^1^				
20–24	1860	12.5 (1.1)	87.5 (1.1)	<0.001
25–34	3430	21.0 (1.0)	79.0 (1.0)	
35–44	3853	29.8 (1.2)	70.2 (1.2)	
45–54	4001	35.7 (1.1)	64.3 (1.1)	
55–64	3057	39.4 (1.2)	60.6 (1.2)	
65–74	3140	39.3 (1.2)	60.7 (1.2)	
75+	2890	30.6 (1.2)	69.4 (1.2)	
Gender				
Female	11,046	34.0 (0.8)	66.0 (0.8)	<0.001
Male	11,185	25.5 (0.7)	74.5 (0.7)	
Race/ethnicity				
Mexican-American	4543	23.4 (0.8)	76.6 (0.8)	<0.001
Other Hispanic	1288	21.8 (1.7)	78.2 (1.7)	
Non-Hispanic white	11,071	33.2 (0.8)	66.8 (0.8)	
Non-Hispanic black	4541	18.8 (0.8)	81.2 (0.8)	
Other race/multi-racial	788	22.5 (1.8)	77.5 (1.8)	
Family income-to-poverty ratio				
0–0.99	3756	19.6 (0.9)	80.4 (0.9)	<0.001
1.0–1.99	5432	23.2 (1.0)	76.8 (1.0)	
2.0–2.99	3347	29.2 (1.4)	70.8 (1.4)	
3.0–3.99	2454	33.3 (1.4)	66.7 (1.4)	
≥4.0	5489	35.8 (1.0)	64.2 (1.0)	
Education (age ≥25)				
<9th grade	3092	17.3 (1.1)	82.7 (1.1)	<0.001
9–11th grade	3257	23.2 (1.1)	76.8 (1.1)	
High-school graduate	4788	29.7 (1.0)	70.3 (1.0)	
Some college	5019	34.0 (1.0)	66.0 (1.0)	
College graduate	3854	37.4 (1.3)	62.6 (1.3)	
Place of birth				
United States	17,253	31.5 (0.6)	68.5 (0.6)	<0.001
Mexico	2669	17.1 (1.1)	82.9 (1.1)	
Elsewhere	2302	19.7 (1.4)	80.3 (1.4)	

^1^ Not age adjusted; LCS: low-calorie sweeteners.

### 3.1. LCS Use and the Healthy Eating Index (HEI 2005)

**Figure 1:**
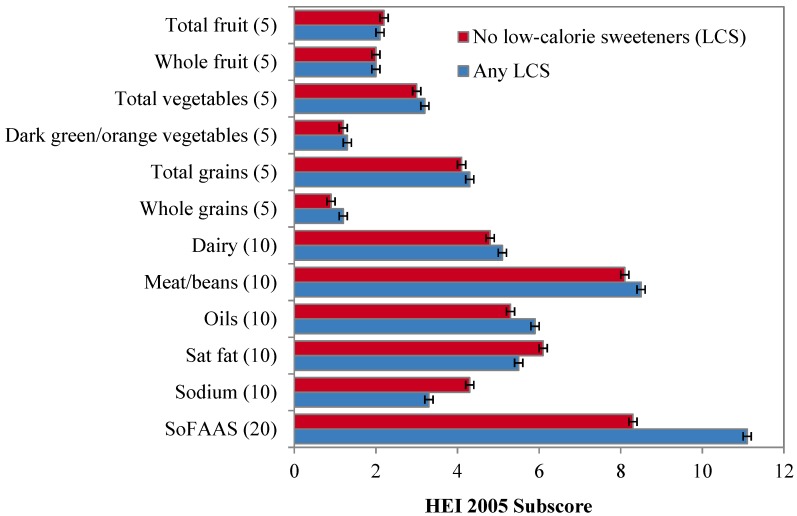
Adjusted association between any low-calorie sweetener (LCS) consumption and mean Healthy Eating Index 2005 (HEI) subscores (maximum value indicated in parentheses). Analysis adjusted for age group, race/ethnicity and gender. Error bars are standard errors.

**Figure 2:**
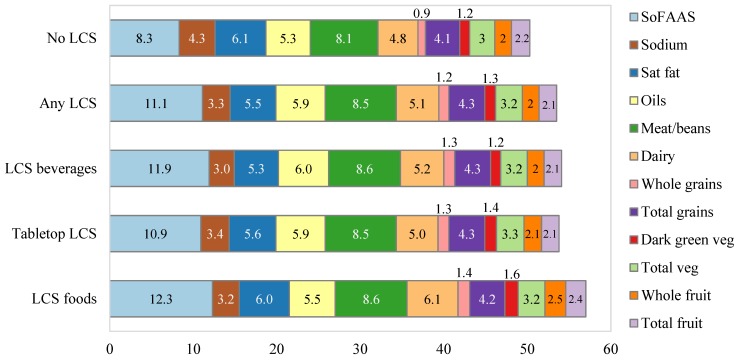
Adjusted mean Healthy Eating Index 2005 (HEI) subscores among non low-calorie sweetener (LCS) users, and LCS use category. Analysis adjusted for age group, race/ethnicity and gender.

Table 2 shows that LCS consumers had significantly higher HEI 2005 scores than did non-consumers, while Figure 1 and Table A1 shows the HEI 2005 subscores for LCS consumers and non-consumers. The association held for consumers of any LCS and for users of LCS beverages, tabletop LCS and for LCS foods (*p* < 0.001) (see Figure 2 and Table A2). The association was largely driven by lower calories from SoFAAS; differences in SoFAAS scores were significant for LCS beverages, for tabletop LCS and for LCS foods (*p* < 0.001). Once energy from SoFAAS were removed from the analysis, the differences in HEI scores were not significant for LCS beverages. However, differences in the total score (minus SoFAAS) were still significant for users of tabletop LCS and LCS foods.

Figure 2 and Table A2 show that users of LCS foods had the highest total HEI-2005 scores. Compared to non-users of LCS foods, this group had significantly higher subscores on total fruit (*p* < 0.004), whole fruit (*p* < 0.001), dark green/orange vegetables and legumes (*p* < 0.001), low fat dairy (*p* < 0.001) and meat and beans (*p* < 0.005). Users of tabletop LCS had significantly higher subscores on total vegetables (*p* < 0.001), dark green/orange vegetables and legumes (*p* < 0.001), whole grains (*p* < 0.001) and meat and beans (*p* < 0.005) compared to non-consumers. Consumers of LCS beverages had higher HEI 2005 subscores for total vegetables (*p* < 0.001), whole grains (*p* < 0.001), milk/dairy (*p* < 0.001) and meat and beans (*p* < 0.001) compared to non-consumers. Consumers of any LCS also tended to have better oils subscore (*i.e.*, higher intake) (*p* < 0.001), though the difference was not significant for LCS foods.

**Table 2:** Adjusted associations between low-calorie sweetener (LCS) use by product category and Healthy Eating Index (HEI 2005) score and SoFAAS ^1^ subscore. Analysis adjusted for age group, gender and race/ethnicity. NHANES 1999–2008.

	HEI 2005	SoFAAS ^1^	HEI (no SoFAAS) ^1^
Any LCS	53.6 (0.3)	11.1 (0.1)	42.5 (0.2)
No LCS	50.4 (0.3)	8.3 (0.1)	42.1 (0.2)
*P*-difference	<0.001	<0.001	0.10
LCS beverages	54.3 (0.4)	11.9 (0.2)	42.3 (0.3)
No LCS beverages	50.7 (0.3)	8.5 (0.1)	42.2 (0.2)
*P*-difference	<0.001	<0.001	0.69
Tabletop LCS	53.8 (0.4)	10.9 (0.2)	42.9 (0.3)
No tabletop LCS	51.1 (0.3)	8.9 (0.1)	42.1 (0.2)
*P*-difference	<0.001	<0.001	0.003
LCS foods	57.1 (0.7)	12.3 (0.3)	44.8 (0.5)
No LCS foods	51.1 (0.2)	9.0 (0.1)	42.1 (0.2)
*P*-difference	<0.001	<0.001	<0.001

^1^ Calories from solid fats, alcohol and added sugars (SoFAAS); LCS: low-calorie sweeteners.

LCS: low-calorie sweeteners.

On the other hand, LCS consumers in every product category had lower HEI subscores for sodium, indicating higher consumption (*p* < 0.001). With the exception of LCS foods, LCS consumers also had lower saturated fat subscores, indicating higher consumption (*p* < 0.001).

### 3.2. LCS Use and Health Behaviors

**Figure 3:**
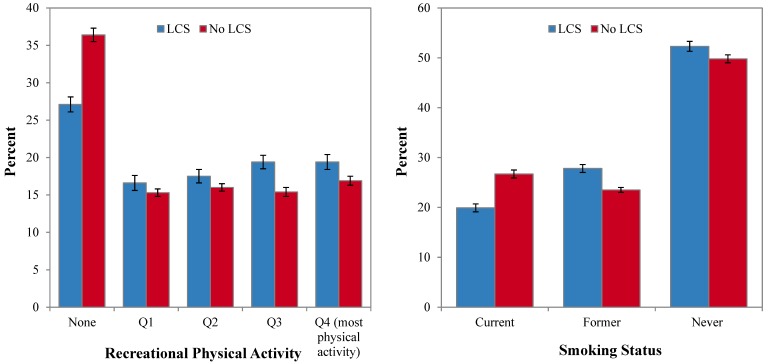
Age-adjusted prevalence of health behaviors among consumers of any low-calorie sweetener (LCS) compared to non-consumers, NHANES 1999–2008. Data are for physical activity (**left**) and for smoking status (**right**).

Figure 3 shows that LCS consumers were more physically active than were non-consumers. The association held after adjusting for age group, gender, family income and race/ethnicity (results not shown). LCS consumers were 15% more likely to be very active compared to non-consumers after adjusting for age only. After adjusting for gender, income and race/ethnicity, LCS consumers were still 10% more likely to be physically active. Those results suggest that the observed link between LCS consumption and higher physical activity cannot be explained simply by differences in SES.

Approximately 20.7% of LCS consumers were current smokers as compared to 26.2% of non-LCS consumers, after adjusting for age group, gender, family income and race/ethnicity. More LCS consumers were former smokers. LCS consumers consumed alcohol less often, though the difference was not profound.

Table 3 shows the health behaviors by LCS product category. LCS beverage consumers were least likely to be sedentary and were more likely to be highly active than were non consumers (in the third or fourth quartiles of Metabolic Equivalent of Task (MET) values for physical activity). LCS beverage consumers were less likely to be current smokers than were non consumers; however, they were more likely to be former smokers. LCS beverage consumers tended to consume less alcohol than did non consumers. Generally, the relation between LCS beverage consumption and health behaviors was similar for both men and women (data not shown).

LCS: low-calorie sweeteners.

**Table 3:** Age-adjusted prevalence (or means) of health behaviors among consumers of low-calorie sweetener (LCS) beverages, tabletop LCS and LCS foods, NHANES 1999–2008.

	*n*	LCS beverages	No LCS beverages	*p*-value	Tabletop LCS	No tabletop LCS	*p*-value	LCS food	No LCS food	*p*-value
Recreational moderate/vigorous physical activity ^1^										
None	6630	26.2 (1.2)	35.5 (0.8)	<0.001	26.6 (1.5)	34.4 (0.8)	<0.001	23.2 (2.1)	34.3 (0.8)	<0.001
Q1	2359	17.3 (1.5)	15.4 (0.4)		15.6 (1.2)	15.7 (0.5)		15.0 (2.1)	15.7 (0.5)	
Q2	2377	18.2 (1.2)	16.1 (0.5)		17.8 (1.5)	16.3 (0.4)		17.9 (2.2)	16.3 (0.4)	
Q3	2370	19.3 (1.1)	16.0 (0.6)		18.2 (1.4)	16.5 (0.5)		22.7 (2.5)	16.5 (0.5)	
Q4 (most physical activity)	2385	19.1 (1.2)	17.0 (0.6)		21.8 (1.9)	17.2 (0.6)		21.3 (2.8)	17.2 (0.6)	
Smoking status										
Current smoker	4997	18.7 (1.0)	26.0 (0.7)	<0.001	20.7 (1.6)	25.2 (0.6)	<0.001	15.1 (1.8)	25.1 (0.6)	<0.001
Former smoker	5861	28.4 (1.2)	24.0 (0.5)		27.7 (1.2)	24.3 (0.4)		25.8 (2.1)	24.8 (0.4)	
Never smoker	11,349	52.9 (1.3)	50.0 (0.7)		51.6 (1.6)	50.5 (0.7)		59.1 (2.6)	50.2 (0.7)	
Frequency of alcohol consumption										
None or <12 drinks in past year	7477	28.1 (1.4)	29.9 (1.1)	0.045	26.8 (1.4)	29.7 (1.2)	<0.001	29.2 (2.6)	29.6 (1.1)	<0.001
<0.2 drinks/wk	3504	16.6 (0.8)	16.3 (0.5)		18.1 (1.5)	16.2 (0.4)		18.8 (1.8)	16.3 (0.4)	
0.2–0.99 drinks/wk	3226	18.8 (1.0)	16.3 (0.4)		17.6 (1.6)	16.6 (0.4)		17.7 (2.0)	16.6 (0.4)	
1–5.99 drinks/wk	3391	19.8 (1.3)	18.3 (0.6)		19.5 (1.6)	18.5 (0.6)		20.1 (2.6)	18.6 (0.6)	
≥6 drinks/wk	3440	16.7 (1.1)	19.2 (0.6)		18.1 (1.8)	19.0 (0.6)		14.2 (1.7)	18.9 (0.8)	
Grams of alcohol (mean) ^2,3^	-	11.2 (0.6)	10.7 (0.4)	0.64	10.2 (0.6)	10.8 (0.4)	0.29	9.4 (0.8)	10.8 (0.4)	0.08

^1^ Data from 1999–2006 NHANES, since Metabolic Equivalent of Task (MET) values were not collected in the 2007–2008 cycle of NHANES. ^2^ Value represents the mean. ^3^ From 24-hour recall; adjusted for energy, gender and age group; LCS: low-calorie sweeteners.

Tabletop LCS consumers were more physically active and were less likely to be current smokers than were non-consumers. Tabletop LCS consumers did not differ in alcohol intakes from non-consumers, though they were slightly less likely to be never or infrequent alcohol drinkers. Similar trends were obtained for consumers of LCS foods. LCS food consumers were much less likely to be current smokers than were non-consumers.

## 4. Discussion

The present study extends prior research by providing the first analyses of the association of LCS use with diet quality measures based on the Healthy Eating Index 2005. Past authors [5] have called for further research on the impact of diet beverages on diet quality, with particular attention to sugar consumption [4,5]. One concern has been that the use of LCS beverages was likely to be associated with higher, as opposed to lower, sugar consumption and with lower-quality diets [4].

The present findings were that LCS users had significantly higher quality diets than did non-users. That observation held for every LCS product category. The improvement in the total HEI 2005 scores was largely driven by more favorable SoFAAS subscores. The SoFAAS component tracks the consumption of solid fats, added sugars and alcohol, the principal sources of “empty calories” [21] in the American diet. For every product category, LCS use was associated with higher SoFAAS subscores, indicating lower consumption of solid fats, alcohol and added sugars.

Additional analyses (Table 3) showed that LCS use was not systematically associated with amount of alcohol consumed from a 24-h recall. Amounts of alcohol consumed were comparable across LCS users and non-users for every LCS product category. The consumption of saturated fat was moderately elevated among LCS consumers. The conclusion is that improved SoFAAS subscores were largely driven by a drop in added sugars consumption among LCS users as opposed to non-users.

The present study used Federal measures of diet quality to counter some interpretations of past research [5]. In one study [5], researchers compared the consumption of selected sweet snacks, such as ice cream, dairy desserts, sweet rolls, cakes, pastries, cookies, pies and candy (696 items) by users of sugar sweetened (SSB) and diet beverages. The observation that both groups derived approximately 11%–12% of their energy intake (238 kcal/day) from sweet snacks was interpreted to mean that the sweet taste of LCS enhanced appetite and encouraged sugar craving and dependence [5,22]. The present analyses do not support that interpretation.

In this study, diet quality was measured using HEI-2005, a 12-component tool designed to measure compliance with dietary recommendations and guidelines. As expected, LCS consumers had more favorable SoFAAS subscores, a measure specifically designed to track the consumption of empty calories. The present data clearly showed that SoFAAS subscores applied to the total diet were significantly higher for LCS users than for non-users, across all product categories, and the association held upon adjustment for covariates. The present conclusion is that the amount of added sugar in the diets of LCS users was, in fact, lower. In general, the consumption of added sugars in U.S. diets has decreased [23,24], whereas the consumption of LCS has increased [14].

Consumers of LCS foods showed the greatest differences in HEI scores. Though fewer adults consumed LCS foods, the difference in HEI scores between consumers and non-consumers was greaterthan for LCS beverages or for tabletop LCS. Consumers of LCS foods had HEI scores that were about 12% higher than non-consumers. Consumers of LCS foods also tended to consume more fruits, vegetables, whole grains, meat/beans and fewer calories from SoFAAS. These data suggest that consumers of LCS foods may be more likely to adopt a prudent diet when compared to consumers of other LCS products.

LCS consumers were less sedentary, exercised more and smoked less than did non-consumers. These positive health behaviors were observed for all product categories: tabletop sweeteners, diet beverages and low calorie foods. One possible explanation is that LCS use may be a proxy marker for higher quality diets and for better health behaviors.

This study had several strengths. First, analyses were based on a large, nationally-representative sample of United States adults. Results are thus generalizable on a population level and can be compared to other recent studies. Second, this was one of the first studies to assign LCS consumers to different product categories. With few exceptions [14], most of the literature has dealt exclusively with diet beverages. Given differences in LCS use by age group and diminishing consumption of carbonated beverages later in life, this classification scheme may improve our understanding of the contextual, behavioral and environmental influences on LCS consumption by SES and demographic variables.

The use of HEI 2005 and its subscores was another strength. Previous studies have called for more research on the overall diet quality of LCS consumers. The HEI 2005 is an accepted metric of diet quality, and has been used in numerous studies evaluating the diet quality of the population. Evaluating diet quality by LCS using alternative metrics is one potential area of future research.

Several limitations should be noted. Owing to the relatively low use of tabletop LCS and LCS foods, the accuracy of consumption data for these products is not well established. Different types of LCS (*i.e.*, sucralose or saccharin) could not be identified in the nutrient composition database. As new LCS foods enter the marketplace, the developers of food composition databases ought to consider the feasibility of explicitly identifying beverages and foods containing LCS.

## 5. Conclusions

The use of diet products, reduced in added sugars, remains a popular strategy for weight control [25], one that has been tested in numerous studies [26,27]. The present analyses suggest that LCS consumers may differ in several, previously unobserved, ways from non-consumers in terms of their health behaviors. In particular, LCS consumers were more physically active and had higher HEI scores. In addition to the benefits conferred by the reduction of energy from added sugars, LCS consumption may be a marker for other positive health behaviors and lifestyles

## Appendix

**Table A1:** Adjusted association between any low-calorie sweetener (LCS) consumption and the Healthy Eating Index 2005 (HEI) score and the HEI components. Analysis adjusted for age group, race/ethnicity and gender.

	Maximum Score	Any LCS	No LCS	*p*-value of Difference
Healthy Eating Index (2005)	100	53.6 (0.3)	50.4 (0.3)	<0.001
HEI 1: Total fruit	5	2.1 (0.1)	2.2 (0.1)	0.08
HEI 2: Whole fruit	5	2.0 (0.1)	2.0 (0.1)	0.38
HEI 3: Total vegetables	5	3.2 (0.1)	3.0 (0.1)	<0.001
HEI 4: Dark green/orange veg and legumes ^1^	5	1.3 (0.1)	1.2 (0.1)	0.15
HEI 5: Total grains	5	4.3 (0.1)	4.1 (0.1)	<0.001
HEI 6: Whole grains	5	1.2 (0.1)	0.9 (0.1)	<0.001
HEI 7: Dairy/milk	10	5.1 (0.1)	4.8 (0.1)	<0.001
HEI 8: Meat and beans	10	8.5 (0.1)	8.1 (0.1)	<0.001
HEI 9: Oils	10	5.9 (0.1)	5.3 (0.1)	<0.001
HEI 10: Saturated fat	10	5.5 (0.1)	6.1 (0.1)	<0.001
HEI 11: Sodium	10	3.3 (0.1)	4.3 (0.1)	<0.001
HEI 12: SoFAAS *	20	11.1 (0.1)	8.3 (0.1)	<0.001
HEI-2005 (no SoFAAS)	80	42.5 (0.2)	42.1 (0.2)	0.10

^1^ Legumes are included in this category if they were not needed to meet the meat and bean component score; * Calories from solid fat, alcoholic beverages and added sugars (SoFAAS); LCS: low-calorie sweetener.

**Table A2:** Adjusted association between any low-calorie sweetener (LCS), tabletop LCS and LCS foods consumption and the Healthy Eating Index 2005 (HEI) score and the HEI components. Analysis adjusted for age group, race/ethnicity and gender.

	Max Score	LCS Beverages	No LCS Beverages	*p*-value	Tabletop LCS	No Tabletop LCS	*p*-value	LCS Foods	No LCS Foods	*p*-value
Healthy Eating Index (2005)	100	54.3 (0.40)	50.7(0.27)	<0.001	53.8 (0.45)	51.1 (0.26)	<0.001	57.1 (0.7)	51.1 (0.2)	<0.001
HEI 1: Total fruit	5	2.1 (0.06)	2.2 (0.04)	0.20	2.1 (0.06)	2.2 (0.04)	0.61	2.4 (0.1)	2.2 (0.04)	0.004
HEI 2: Whole fruit	5	2.0 (0.06)	2.0 (0.04)	0.31	2.1 (0.06)	2.0 (0.03)	0.19	2.5 (0.1)	2.0 (0.04)	<0.001
HEI 3: Total vegetables	5	3.2 (0.04)	3.1 (0.02)	<0.001	3.3 (0.04)	3.1 (0.02)	<0.001	3.2 (0.1)	3.1 (0.02)	0.10
HEI4: Dark green/orange vegetables and legumes ^1^	5	1.2 (0.04)	1.3 (0.03)	0.55	1.4 (0.06)	1.2 (0.03)	<0.001	1.6 (0.1)	1.2 (0.03)	<0.001
HEI 5: Total grains	5	4.3 (0.03)	4.1 (0.02)	<0.001	4.3 (0.04)	4.2 (0.01)	0.01	4.2 (0.16)	4.2 (0.02)	0.80
HEI 6: Whole grains	5	1.3 (0.04)	1.0 (0.02)	<0.001	1.3 (0.05)	1.0 (0.02)	<0.001	1.4 (0.1)	1.0 (0.02)	0.01
HEI 7: Dairy/milk	10	5.2 (0.08)	4.8 (0.05)	<0.001	5.0 (0.10)	4.9 (0.05)	0.13	6.1 (0.2)	4.8 (0.05)	<0.001
HEI 8: Meat and beans	10	8.6 (0.05)	8.1 (0.03)	<0.001	8.5 (0.07)	8.2 (0.03)	<0.001	8.6 (0.1)	8.2 (0.03)	0.005
HEI 9: Oils	10	6.0 (0.08)	5.3 (0.05)	<0.001	5.9 (0.11)	5.4 (0.04)	<0.001	5.5 (0.2)	5.4 (0.04)	0.61
HEI 10: Saturated fat	10	5.3 (0.11)	6.1 (0.05)	<0.001	5.6 (0.11)	6.0 (0.05)	<0.001	6.0 (0.2)	5.9 (0.05)	0.59
HEI 11: Sodium	10	3.0 (0.06)	4.3 (0.04)	<0.001	3.4 (0.09)	4.1 (0.04)	<0.001	3.2 (0.1)	4.1 (0.04)	<0.001
HEI 12: SoFAAS *	20	11.9 (0.18)	8.5 (0.12)	<0.001	10.9 (0.2)	8.9 (0.1)	<0.001	12.3 (0.3)	9.0 (0.10)	<0.001

^1^ Legumes are includes in this category if they were not needed to meet the meat and bean component score; * Calories from solid fats, alcohol and added sugars (SoFAAS); LCS: low-calorie sweetener.
